# Advanced Carbon Nanostructures: Synthesis, Properties, and Applications II

**DOI:** 10.3390/nano14242026

**Published:** 2024-12-17

**Authors:** Marianna V. Kharlamova

**Affiliations:** Centre for Advanced Material Application (CEMEA), Slovak Academy of Sciences, Dúbravská Cesta 5807/9, 845 11 Bratislava, Slovakia; mv.kharlamova@gmail.com

Single-walled carbon nanotubes (SWCNTs) are practiced in various areas, such as nanoelectronics, sensors, photovoltaics, electrochemical energy storage devices, nanobiotechnology, and nanocomposite fillers. Filled carbon nanotubes are more likely to be used for these applications than pure SWCNTs, because they have bigger chemical and physical features that guide functional applications [1,2,3,4,5,6,7,8,9,10]. SWCNTs have unique electronic structures, great conductivity, and high transparency, which suggests applying them as nanoelectronics components. SWCNTs have excellent capability in rapid charge and discharge and long cycling stability, which permits employing them as electrochemical energy storage appliances. Doped SWCNTs are applied as light-emitting p-n diodes with lessened power dissipation and negligible self-heating. SWCNTs have a large Seebeck coefficient, which assumes applying them as thermoelectric stuff. SWCNTs have the capacity to act as interfacial agents or as transparent conductive electrodes in solar cells. A large surface area of SWCNTs affords the ability to load and deliver diagnostic and medical agents to the tissues and organs. SWCNTs are valuable implements for bioimaging and anticancer therapy. The Special Issue “Advanced Carbon Nanostructures: Synthesis, Properties, and Applications II” is dedicated to the novel insights in the physical properties of carbon nanomaterials. The Special Issue includes six papers, and one review article. The papers were devoted to metal-supported and encapsulated carbon nanomaterials for catalytic and sensor applications as well as pristine carbon nanomaterial preparation and investigation. In Ref. [11], Pt nanoparticle-supported graphene aerogel was prepared, and it was tested for catalytic applications. The samples had a 3D porous structure, a large specific surface area, and good electrical conductivity (Figure 1).

In Ref. [12], Fe_3_C-nanoparticle-encapsulated nitrogen-doped hierarchically porous carbon membranes were prepared (Figure 2). The authors developed an efficient method for the colorimetric sensing of ascorbic acid.

In Ref. [13], aerosol-synthesized SWCNT films for silicon nitride photonic circuits were prepared as a basis for developing integrated optics devices (Figure 3) and the optical properties of the samples were revealed.

In Ref. [14], the authors synthesized graphene on an epitaxial single-crystal Cu film deposited and recrystallized on a basal-plane sapphire substrate. The influence of the synthesis parameters on the properties of the samples was investigated and the high quality of the samples was confirmed (Figure 4).

In Ref. [15], authors demonstrated the novel method for directly growing patterned vertical graphene (VG) on a SiO_2_/Si substrate (Figure 5).

In review article [16], the author presented advances in the understanding of the kinetics and electronic properties of filled SWCNTs. Metal, metal halide, metal chalcogenide, and metallocene-filled SWCNTs were discussed and advances in spectroscopic investigations of filled SWCNTs were presented. This is important in SWCNT applications.

We invite all interested scientists to read the articles published in the Special Issue “Advanced Carbon Nanostructures: Synthesis, Properties, and Applications II”. We think that the published articles are useful for scientists from different disciplines.

## Figures and Tables

**Figure 1:**
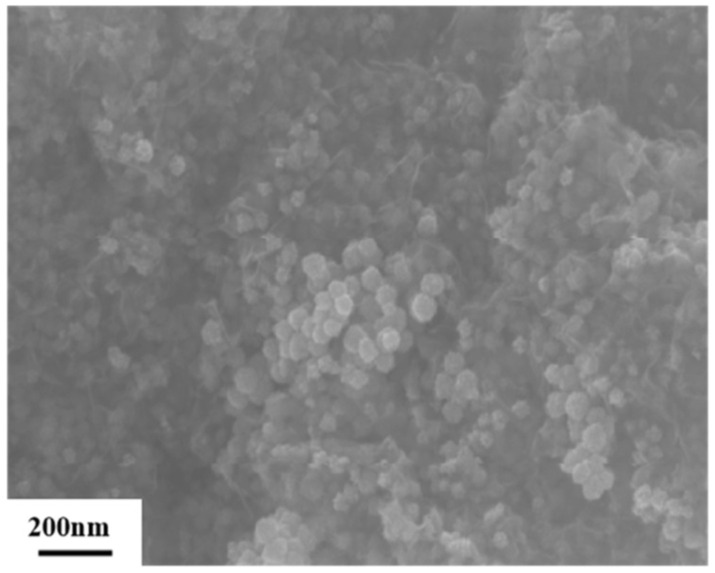
The SEM data of Pt nanoparticle-supported graphene aerogel [11]. Copyright 2024 by the authors. Licensee MDPI, Basel, Switzerland. This article is an open access article distributed under the terms and conditions of the Creative Commons Attribution (CC BY) license.

**Figure 2:**
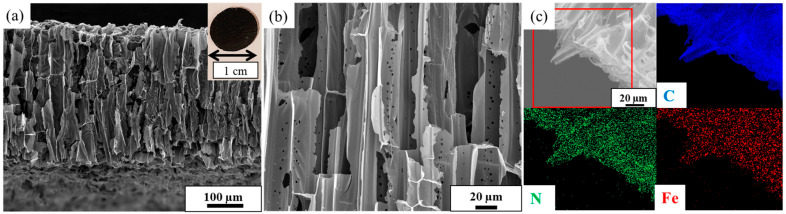
(**a**,**b**) Cross-sectional SEM images of the membranes. The inset in (**a**) is the photograph of the membrane. (**c**) Elemental mapping of different elements in the sample [12]. Copyright 2023 by the authors. Licensee MDPI, Basel, Switzerland. This article is an open access article distributed under the terms and conditions of the Creative Commons Attribution (CC BY) license.

**Figure 3:**
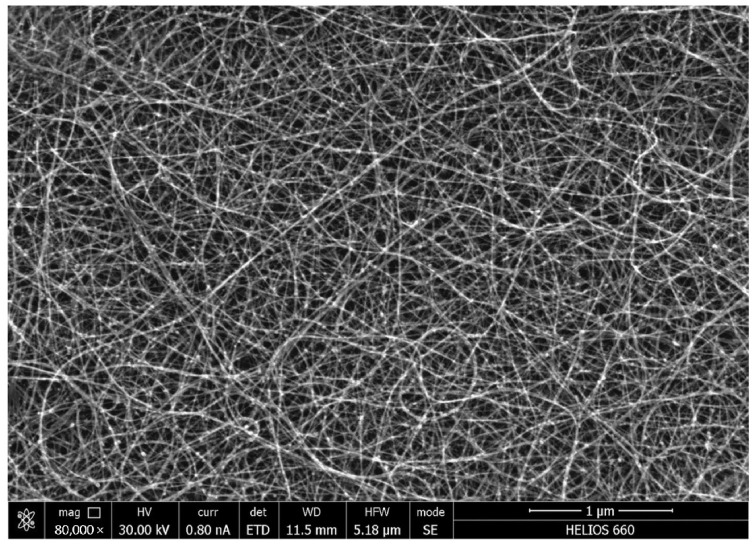
The SEM image of the SWCNT film [13]. Copyright 2023 by the authors. Licensee MDPI, Basel, Switzerland. This article is an open access article distributed under the terms and conditions of the Creative Commons Attribution (CC BY) license.

**Figure 4:**
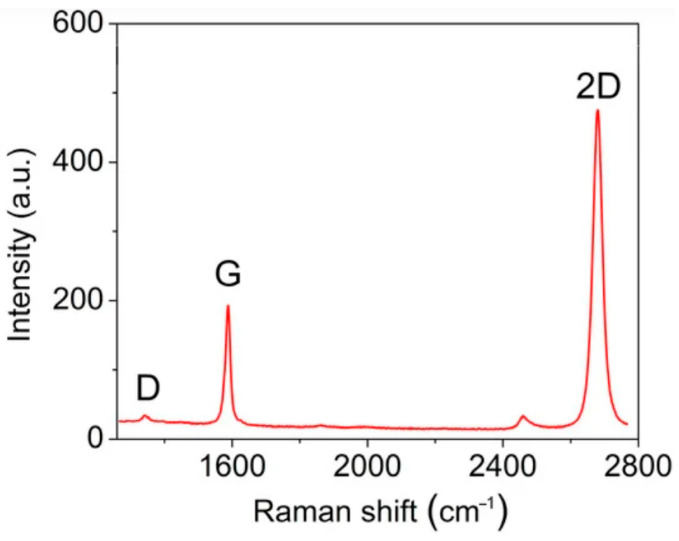
The Raman spectra of graphene synthesized on copper film with thickness d = 2.4 μm [14]. Copyright 2023 by the authors. Licensee MDPI, Basel, Switzerland. This article is an open access article distributed under the terms and conditions of the Creative Commons Attribution (CC BY) license.

**Figure 5:**
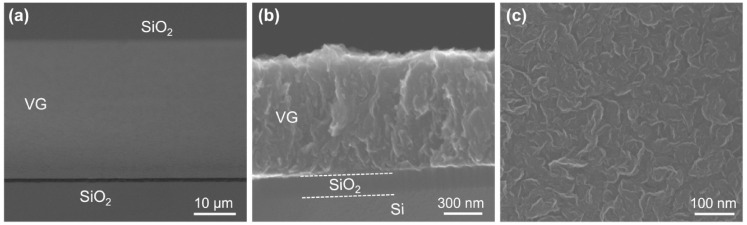
The SEM data. (**a**) The oblique view, (**b**) cross-sectional view, and (**c**) top view of the patterned VG grown directly on a SiO_2_/Si substrate [15]. Copyright 2023 by the authors. Licensee MDPI, Basel, Switzerland. This article is an open access article distributed under the terms and conditions of the Creative Commons Attribution (CC BY) license.

## Data Availability

Data are available on request from the first author (Marianna V. Kharlamova).
